# Two-dimensional nanosecond electric field mapping based on cell electropermeabilization

**DOI:** 10.1186/1757-5036-2-9

**Published:** 2009-11-11

**Authors:** Meng-Tse Chen, Chunqi Jiang, P Thomas Vernier, Yu-Hsuan Wu, Martin A Gundersen

**Affiliations:** 1 Mork Family Department of Chemical Engineering and Materials Science, University of Southern California, Los Angeles, CA 90089, USA; 2 Ming Hsieh Department of Electrical Engineering-Electrophysics, University of Southern California, Los Angeles, CA 90089, USA; 3 MOSIS, Information Sciences Institute, University of Southern California, Marina del Rey, CA 90292, USA

## Abstract

Nanosecond, megavolt-per-meter electric pulses cause permeabilization of cells to small molecules, programmed cell death (apoptosis) in tumor cells, and are under evaluation as a treatment for skin cancer. We use nanoelectroporation and fluorescence imaging to construct two-dimensional maps of the electric field associated with delivery of 15 ns, 10 kV pulses to monolayers of the human prostate cancer cell line PC3 from three different electrode configurations: single-needle, five-needle, and flat-cut coaxial cable. Influx of the normally impermeant fluorescent dye YO-PRO-1 serves as a sensitive indicator of membrane permeabilization. The level of fluorescence emission after pulse exposure is proportional to the applied electric field strength. Spatial electric field distributions were compared in a plane normal to the center axis and 15-20 μm from the tip of the center electrode. Measurement results agree well with models for the three electrode arrangements evaluated in this study. This live-cell method for measuring a nanosecond pulsed electric field distribution provides an operationally meaningful calibration of electrode designs for biological applications and permits visualization of the relative sensitivities of different cell types to nanoelectropulse stimulation. PACS Codes: 87.85.M-

## 1. Introduction

Ultra-short (< 100 ns), high-field (MV/m) electric pulses produce a variety of effects [1], including release of intracellular calcium [2,3], eosinophil disruption [4], vacuole permeabilization [5], mitochondrial release of cytochrome c [6], caspase activation [7,8], and phosphatidylserine (PS) externalization [9,10]. Nanosecond electric pulses have been shown to kill a wide variety of human cancer cells in vitro, including basal cell carcinoma and pancreatic cancer cells, and to induce tumor regression in vivo [11,12], and nanoelectropulse therapy is under development for skin cancer treatment. Some studies of nanosecond pulse effects on tumors have been carried out with parallel-plate electrodes, like those in commercial electroporation cuvettes, where fringing effects are negligible and the electric field distribution can be assumed to be homogeneous. In published [11,12] and ongoing efforts directed at tumor therapy, however, needle-array electrodes are employed, for which the electric field distribution is not as simple. Magnetic resonance current density imaging and three-dimensional finite modeling were employed to qualitatively evaluate the electric field distribution of different electrode configurations in a prior study of in vivo electroporation [13]. In the present work we demonstrate, using live cell responses, a qualitative mapping of the electric field around three electrode configurations, and we show the correspondence of these electric field profiles with those expected from electromagnetic modeling. Extension of this method can lead to a better and more rigorously quantitative analysis of electric field distributions around electrodes in biological systems, leading to an increased understanding of the in vivo electroporation process and also contributing to evaluations of the efficacy of nanoelectropulse exposure in clinical applications.

In this paper we report the use of living cell monolayers as nanoelectroporation-based, two-dimensional electric field sensors. Fluorescence imaging patterns from the pulse-induced influx of YO-PRO-1 are used to construct two-dimensional maps of the electric field applied with three electrode assemblies -- single-needle, five-needle array, and flat-cut coaxial cable -- immersed in biological media over the monolayers. The field distributions from the different electrode configurations and the responses of different types of cells to nanosecond pulses are compared. In addition, finite element method-based software, COMSOL Multiphysics, was used to calculate the electric field distribution for an electrostatic model. Modeling results and measurements are compared.

## 2. Materials and Methods

### 2.1 Experimental setup

**Figure 1:**
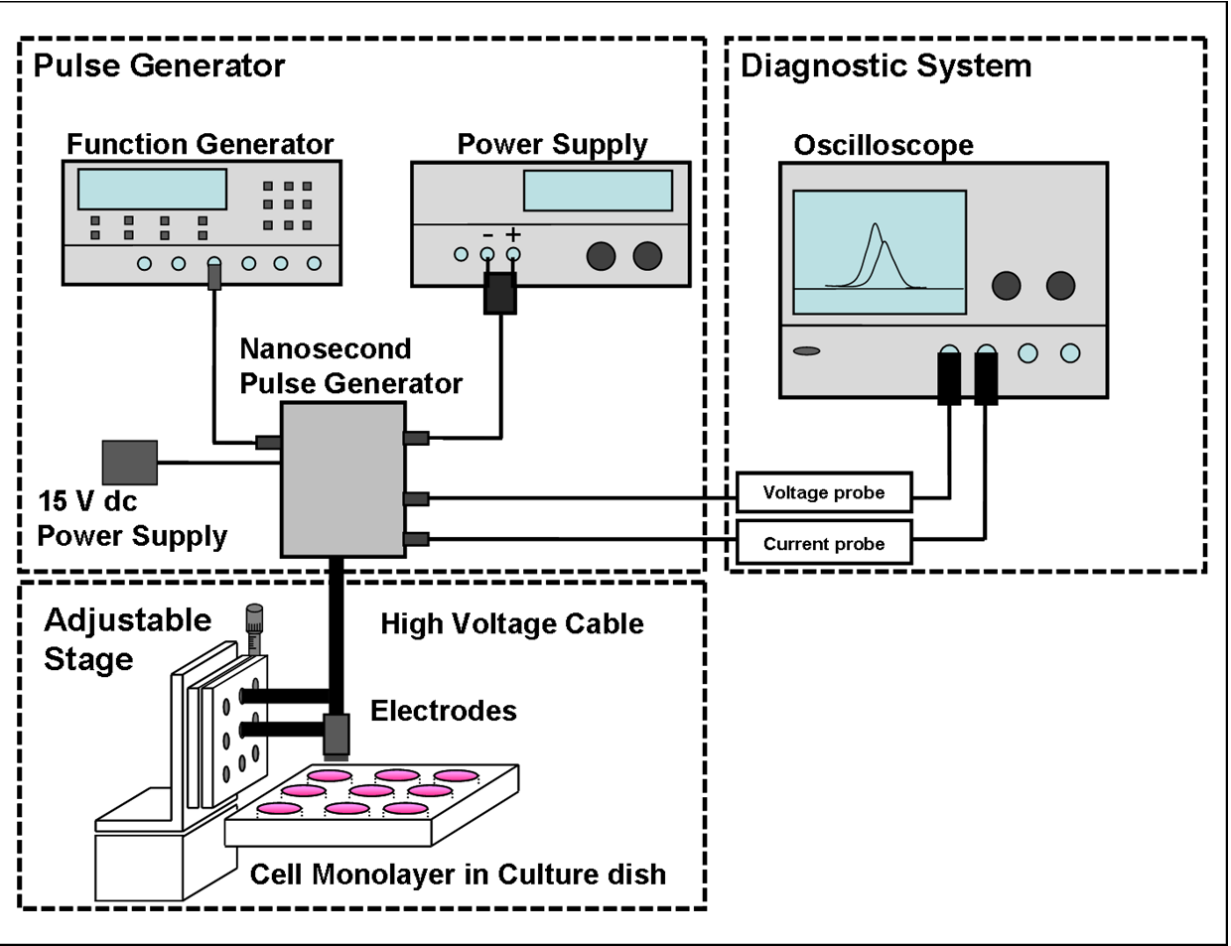
**Schematic of the experimental setup for nanosecond pulsed electric field mapping**.

The experimental setup consists of a pulse generator, a voltage and current diagnostic system, and an optical stage for accurately positioning a cell culture plate, as shown in Figure 1.

#### 2.1.1 Pulse generation and measurement

A solid-state, opening-switch-based pulse generator, generating 15 ns, 10 kV pulses at repetition rates up to 50 Hz, was designed and fabricated at the University of Southern California [14]. A built-in resistive voltage divider based on cascaded attenuation stages with a total attenuation of -54 dB (1:500) was used to measure the pulse voltage delivered to the load [15]. A current transformer with a ratio of 1 to 5 was used to measure the pulse current. A high saturation flux density Finemet^® ^Metglas core (ID = 0.8 cm, OD = 1.5 cm, h = 0.6 cm) provides fast response and linearity for the current measurement. The attenuated pulse current was converted to a voltage signal with a 50 ohm, surface-mount, low-inductance resistor, terminated at the secondary winding of the transformer, to give a total current-to-voltage conversion of 20 V/A. A 50 ohm-terminated digital oscilloscope (Tektronix TDS 5104) was connected with 50 ohm coaxial cables to record the output from the voltage divider and the current sensor.

A 50 ohm SHV coaxial cable assembly was used to deliver nanosecond electric pulses to the electrodes. The losses in the cable are about 3%, single transit. Since the pulse generator represents an open circuit for the reflected pulses and the impedance of the electrodes is 100 ohms or less, depending on the electrode configuration, the reflected pulse amplitude is always less than 50%. Because of the complexity and variable impedance of biological loads precise matching is not possible, but since nanosecond biolectric effects are primarily dependent on the applied electric field, power transfer is not a critical consideration.

#### 2.1.2 Electrode configurations

**Figure 2:**
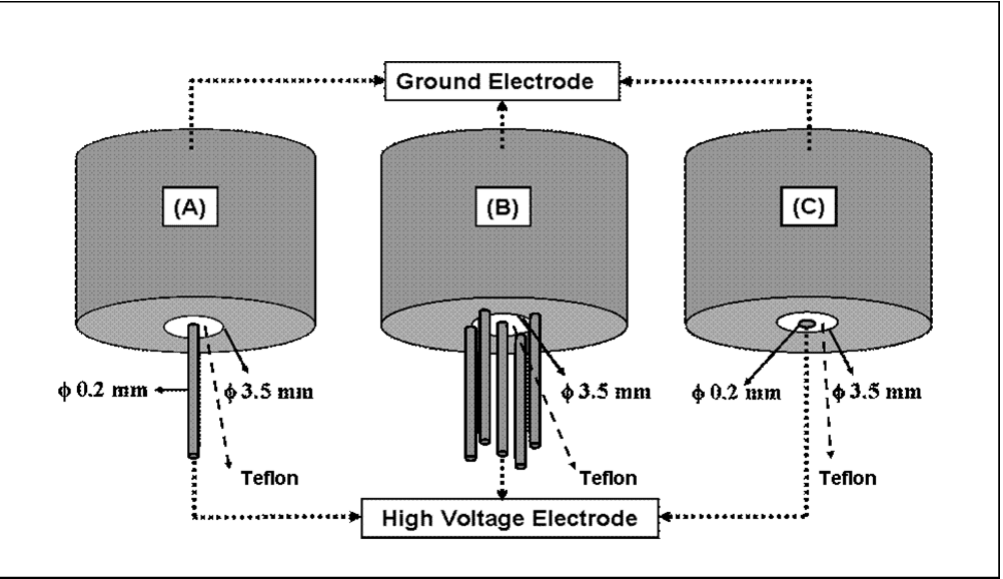
**Three types of electrode configurations designed for nanoelectropulse treatment and cancer therapy**. (A) single-needle, (B) five-needle array, and (C) flat-cut cable.

Three types of electrode assemblies, single-needle, five-needle, and a flat-cut coaxial cable, as shown in Figure 2, were tested. All the electrodes are in center-symmetrical configurations. Stainless steel needles, 0.2 mm in diameter and 5 mm long, are used for the single-needle and five-needle electrodes. The needle tips are cut off and the edges rounded by polishing, leaving a cylindrical profile. For the five-needle array, one needle is at the center, and the other four needles, equally spaced, are located on a 3.5 mm-diameter circle and laser-welded to the coaxial outer shield. For the flat-cut cable, the center stainless steel electrode is 0.2 mm in diameter, and the end surfaces of the electrodes and the insulator are in the same plane. The separation between center and outer conductors is 1.7 mm. The insulating material separating the center electrode and the outer grounding shield is Teflon. The other end of these electrodes is an SHV adapter to facilitate connections with SHV cable assemblies.

#### 2.1.3 Adjustable stage

An adjustable stage incorporating a screw-driven micrometer with a resolution of 25 μm was used to adjust the distance between the cells and the electrodes. The culture dish containing the experimental samples was placed on a rigid, level horizontal surface. The electrode assembly was fixed to an arm of the mechanical stage, and the center electrode distance was adjusted with the center axis normal to the bottom surface of the culture dish. The cell monolayer has a thickness of 5-10 μm. After identifying the location where the electrodes touch the bottom of the culture dish, we retracted the electrodes upward 25 μm to obtain a monolayer-to-electrode tip spacing of 15-20 μm.

### 2.2 Cell lines and cell preparations

Human Jurkat T lymphoblasts (ATCC TIB-152) were cultured in suspension with RPMI 1640 medium (Irvine Scientific, Irvine, CA) containing 10% heat-inactivated fetal bovine serum (FBS; Gibco, Carlsbad, CA), 2 mM L-glutamine (Gibco), 45 units/mL penicillin (Gibco), and 45 μg/mL streptomycin (Gibco). Human prostate cancer PC3 cells (ATCC CRL-1435), U251 human glioblastoma cells (RCB-0461, RIKEN CELL BANK), and human keratoacanthoma cells (skin, mixed morphology, ATCC CRL-7630) were grown in Dulbecco's Modified Eagle's Medium (DMEM, ATCC) with 4 mM L-glutamine, 4500 mg/L glucose, 1 mM sodium pyruvate, 1500 mg/L sodium bicarbonate, 10% FBS, 45 units/mL penicillin, and 45 μg/mL streptomycin. All cells were grown at 37°C in a humidified, 5% CO_2 _atmosphere. Before an experiment, the PC3, U251, and keratoacanthoma cells were detached with 0.05% trypsin/0.53 mM EDTA in Hank's Buffered Salt Solution (HBSS) without sodium bicarbonate, calcium and magnesium (Cellgro, Herndon, VA) and washed with DMEM growth medium. 1 mL of PC3, U251, and keratoacanthoma cell suspensions (1 × 10^6 ^cells/mL) was added to appropriate flat-bottomed wells of a 24-well culture plate, and the cells were incubated until they reached confluence (about 24 hours).

### 2.3 Fluorescence microscopy and imaging processing

YO-PRO-1 (Molecular Probes, Invitrogen; λ_ex _= 491 nm, λ_em _= 509 nm) is a membrane-impermeant fluorescent probe. A permeabilized cell can be identified by the greatly increased fluorescence resulting from YO-PRO-1 influx and binding to nucleic acid material in the cell interior. A Zeiss AxioVert 200 M fluorescence microscope (Carl Zeiss Micro Imaging, Inc., Thornwood, NY) and AxioVision 3.1 imaging software were used to capture and analyze fluorescence images. Low-power (10× objective) images of cell monolayers were taken 15 minutes after pulse exposure. Since the total area of the pulse-exposed cells is greater than the imaging region of the 10× objective, composite images were generated from a sequence of overlapping images that covered the entire area under and around the electrodes. Each experiment was performed three times, with similar results in each case.

### 2.4 Electrostatic calculation of the electric field distribution

The wavelength, ***λ***, and the skin depth, ***d***, of an electromagnetic wave propagating in a medium with zero magnetic susceptibility (relative permeability = 1) depend on the frequency, ***f***, and on the dielectric constant, ***ε***_*r*_, and conductivity, ***σ***, of the medium, as in the following equations (1) and (2):

and

where *c *is the speed of light in vacuum, **ω = 2πf **is the angular frequency, and *ε *is the permittivity of free space. The rise and fall times of the 15 ns voltage pulses are 5 ns or longer so that the primary frequency of the pulses is expected to be 200 MHz or lower. For DMEM (approximate conductivity: *σ *= 1.4 S/m, and dielectric constant: *ε*_*r *_≈ 80 [16]), the minimum wavelength and the skin depth of the electromagnetic fields are 14 cm and 4 cm, respectively. Since both the wavelength and the skin depth are large compared to the geometry of interest, we can use an electrostatic model, as implemented in the electrostatics module of COMSOL Multiphysics http://www.comsol.com/, for the electric field distribution calculation.

### 2.5 Temperature measurement

**Figure 3:**
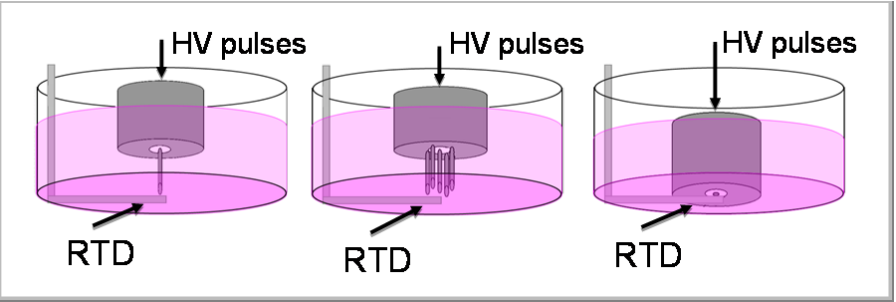
**Temperature measurement setups at the tips of single-needle, five-needle array, and flat-cut cable electrodes powered with continuous electric pulses (10 kV, 15 ns @ 50 Hz)**.

To evaluate the possibility of thermal effects caused by nanosecond electric pulses, we have conducted experiments to measure the localized temperature change at the tips of electrodes with a platinum resistance temperature detector (RTD) (OMEGA, HSRTD-3-100-A-40-E). The hermetically sealed and electrically insulated RTD was placed on the bottom of a 6-well culture plate, and the electrode tips were adjusted until they barely contacted the RTD protective coating. DMEM solution (5 mL) was added to cover the RTD and the electrode tips, as shown in Figure 3.

## 3. Results and Discussion

### 3.1 Nanoelectropulse-induced membrane permeabilization depends on pulse amplitude

**Figure 4:**
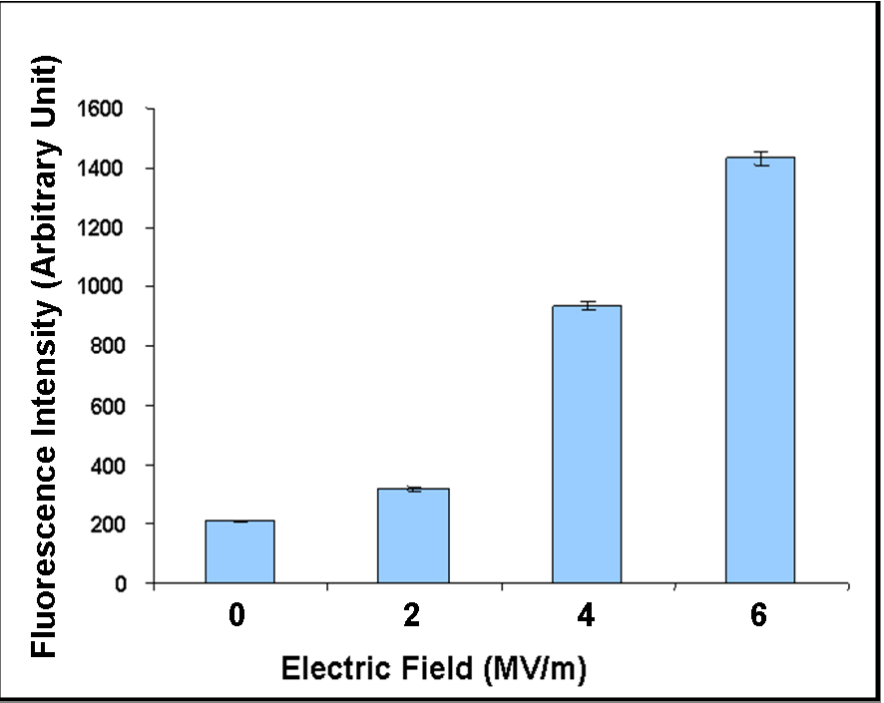
**YO-PRO-1 permeabilization of Jurkat T lymphoblast cells exposed to nanoelectropulses with different electric field amplitudes**. Fluorescence microscopic images were captured 15 minutes after Jurkat cells in growth medium containing YO-PRO-1 (1.0 μM) were exposed to 50, 15 ns pulses at 50 Hz with electric field values of 0, 2, 4 and 6 MV/m. The fluorescence intensity change for each condition was measured by photometric integration. Each data point is from at least 300 representative cells from three independent experiments. Error bars represent the standard error of the sample mean.

It has been reported that cellular permeabilization in Jurkat T lymphoblasts with ultra-short (< 10 ns), high-field (MV/m) electric pulses is a function of pulse count [17]. To quantify the YO-PRO-1 uptake of cells in suspension exposed to nanoelectropulses at different electric field amplitudes, pulses were delivered to Jurkat cells in standard electroporation cuvettes with a 1 mm electrode gap. Cells (10^7 ^cells/mL) in growth medium containing 1 μM YO-PRO-1 were exposed to 50, 15 ns pulses at 50 Hz with electric field intensities of 0, 2, 4, and 6 MV/m. Treated cells were transferred to 8-well cover glass chambers. After 15 minutes fluorescence images of the cells were generated with the 20× objective. Experiments were repeated twice, with more than 1000 cells analyzed for integrated fluorescence intensity for each test condition. The results are summarized in Figure 4, which shows that cell permeabilization to YO-PRO-1 depends on the magnitude of the applied pulsed electric field. In experiments in which other variables are held constant, YO-PRO-1 fluorescence intensity is an indicator of the strength of the local electric field.

### 3.2 Electrical measurement

**Figure 5:**
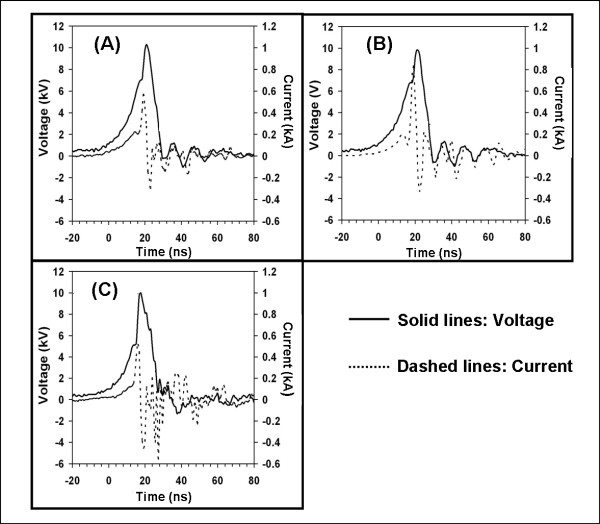
**Typical voltage and current pulse waveforms delivered to the PC3 cell monolayer in DMEM growth medium with three types of electrode configurations**. (A) single-needle, (B) five-needle array, and (C) flat-cut cable.

Nanoelectropulses were applied with each of the three electrode configurations to cell monolayers, with an electrode-cell spacing distance of about 25 μm. Typical pulse voltage and current waveforms are shown in Figure 5. Different peak currents -- 900 A, 600 A, and 500 A -- were observed for the five-needle, single-needle, and flat-cut cable electrodes, respectively. The observed current pulses are a summation of the displacement current associated with the capacitance of the electrodes and the resistive current due to charge migration in the medium. The energy per pulse was calculated by integrating the product of the voltage and current pulse waveforms over a complete pulse period. The energy per pulse for the flat-cut cable, 1.5 mJ, is ten times less than that for the single-needle electrode, 19.1 mJ, and the five-needle array, 20.5 mJ. Thus the pulse energy delivered to the biological load can vary over a wide range, depending on the electrode configuration.

### 3.3 Electric field mapping

#### 3.3.1 Fluorescence images of three electrode configurations

**Figure 6:**
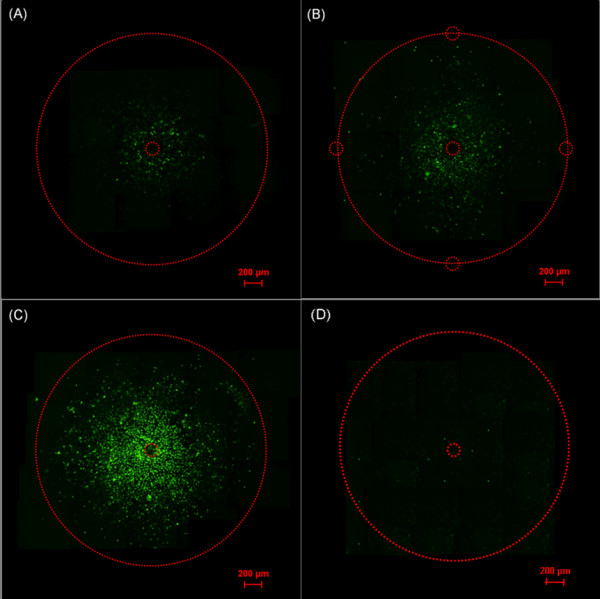
**Fluorescence images of the PC3 cell monolayer exposed to nanoelectropulses with three different electrode configurations, (A) single-needle, (B) five-needle array, and (C) flat-cut cable, based on YO-PRO-1 permeabilization**. 1000, 15 ns, 10 kV pulses at 50 Hz were delivered to cell monolayer. (D) Fluorescence image of un-pulsed PC3 cell monolayer with flat-cut cable as CTRL sample. To generate the fluorescence images of the area between the ground and high voltage electrodes, a series of 640 μm × 710 μm images are combined. The needles and the inner boundary of the ground electrodes are indicated with a small red dashed circle and outer red dashed circles, respectively.

PC3 cell monolayers were exposed to nanoelectropulses (1000, 15 ns, 10 kV pulses at 50 Hz) with three different electrode configurations in fresh DMEM containing 1 μM YO-PRO-1. To rule out effects of mechanical damage resulting from electrode contact, control samples were treated exactly the same as experimental samples, with electrodes adjusted to the same distance, but without actually delivering the pulses to the cells. Figure 6 panels (A), (B), and (C) show fluorescence images of permeabilized PC3 cells after nanoelectropulse exposures with the single-needle, five-needle, and flat-cut cable electrode configurations, respectively. No YO-PRO-1 uptake was observed in control cells (Figure 6(D)). The fluorescence patterns for each image indicate the extent of YO-PRO-1 permeabilization and the affected area of cells for each electrode arrangement. The concentric shape of the fluorescence pattern from cells exposed to the single-needle and flat-cut cable electrodes corresponds to the geometry of the electrode configuration (Figure 6(A) and 6(C). The brightness of the fluorescence pattern for the five-needle array lies between the brightness of the single-needle and flat-cut cable electrode patterns. A square-shape fluorescence pattern centered at the center needle electrode is discernible for the five-needle array. The slight asymmetry and irregularities may result from factors such as the planarity of the cell monolayer and co-planarity of the electrode surfaces In order to distinguish the darker center region and the surrounding brighter regions, the brightness and contrast were adjusted with the imaging software. Therefore, the center needle was located by determining the dark round area with a diameter 0.2 mm. The fluorescence intensity is strongest near the center conductor of the flat-cut cable electrode. The five-needle array produces fluorescence of intermediate intensity; the single-needle has the weakest effect.

#### 3.3.2 Comparisons of electric field distribution between electrostatic simulation and fluorescence integration analysis

Three-dimensional electrostatic models for each of the three electrode configurations (Figure 2) immersed in water were generated, considering the electrodes to be perfect conductors. Gauss's law, **-∇·ε_r_∈_0_∇V = ρ **(with **ρ **= **0**), where V is the electric potential, was solved for a 1 V potential difference between the center electrodes and the ground electrodes. Zero space charge and zero electric displacement were assumed at the dielectric boundaries.

**Figure 7:**
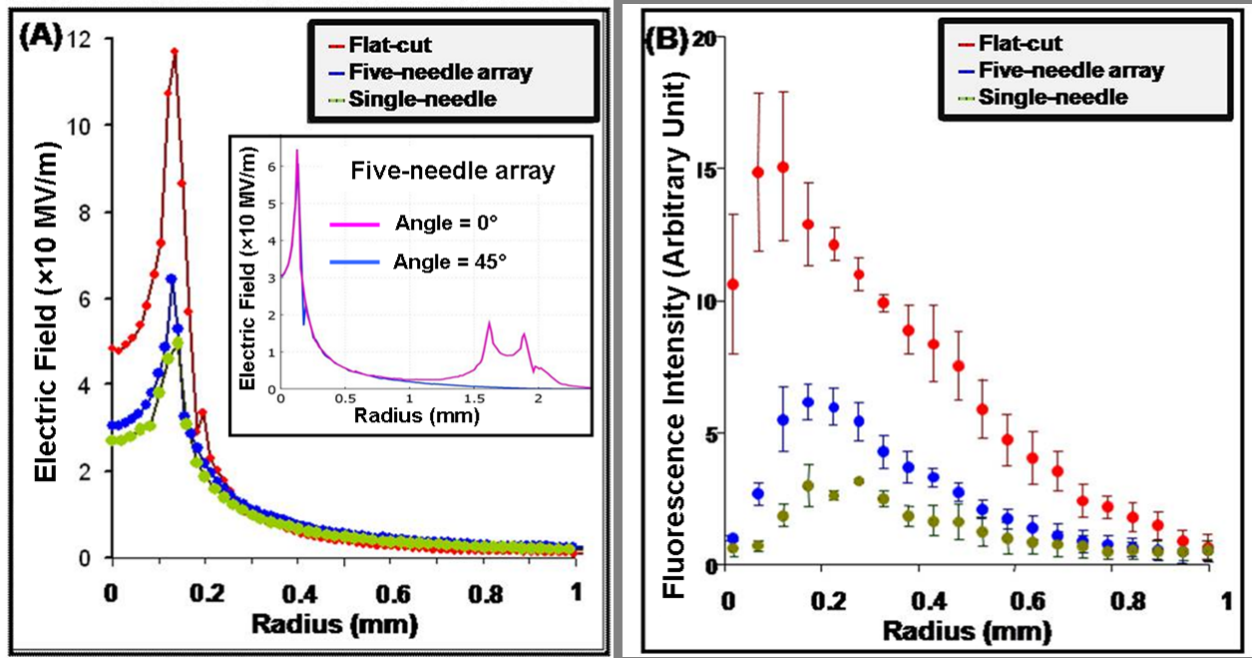
**Electric field distributions modeled with the COMSOL Multiphysics electrostatics module (A) and fluorescence intensity distributions of electropermeabilized PC3 cell monolayers (B) for three different electrode configurations**.

The radial distributions of the electric field for the three electrode configurations in a plane perpendicular to the center axis and 10 μm above the center electrodes are shown in Figure 7(A). The flat-cut cable electrode has a higher electric field at the center of the exposure plane than the five-needle and single-needle electrodes. The maximum electric field appears at the edges of the center electrodes for all the electrode configurations. In the regions beyond the perimeter of the center conductors, the electric field decays rapidly with the radial distance. When 10 kV electric pulses are applied to the center electrodes, the electric field decays to <10 MV/m at a radius of 0.4 mm for all three electrode configurations. Note that the five-needle array is not cylindrically symmetrical. For a given radius the field at a point closer to a ground (outer) electrode will be greater than the field at a point farther away. To make this profile more clear, we define a radial line connecting the center needle with one of the ground needles at an angle of zero, at angle of 45°, the radial line is cross the middle of two ground needles. The electric fields of the five-needle array along radius at angle = 0° and 45° are compared, as shown in the insert plot of Figure 7(A), and implies minimum difference in the field intensity for a radial distance <1 mm.

To better compare the calculated electric field distribution with the measured permeabilization effect, the radial distribution of the relative fluorescence intensities for the three electrode configurations are shown in Figure 7(B). The relative fluorescence intensity was calculated by averaging the total intensity over 10 μm wide concentric rings spaced 50 μm apart. The integration was done in a full concentric circle within 1 mm radius. Again, the flat-cut cable electrode shows a higher fluorescence intensity at the center of the electrode than the other two electrodes. All three electrode configurations have a maximum fluorescence intensity near the edge of the center electrode (r = 0.125 mm). These results agree with the simulated behavior of the electric field distributions with these electrode configurations. For each electrode configuration, Table 1 shows the maximum applied electric field intensity and the maximum fluorescence intensity of permeabilized PC3 cell monolayers.

**Table 1:** Maximum electric field intensity of electrostatic simulation and the maximum fluorescence intensity of permeabilized PC3 cell monolayers.

Electrode Configurations	Maximum Electric Field Intensity (MV/m)	Maximum Fluorescence Intensity (Arbitrary Unit)
Single needle electrode	5.0	3.3 ± 0.8
Five-needle array electrode	6.5	6.5 ± 0.8
Flat-cut cable	11.5	15.1 ± 3.0

#### 3.3.3 Fluorescence images of various cell types at different spacing distances

**Figure 8:**
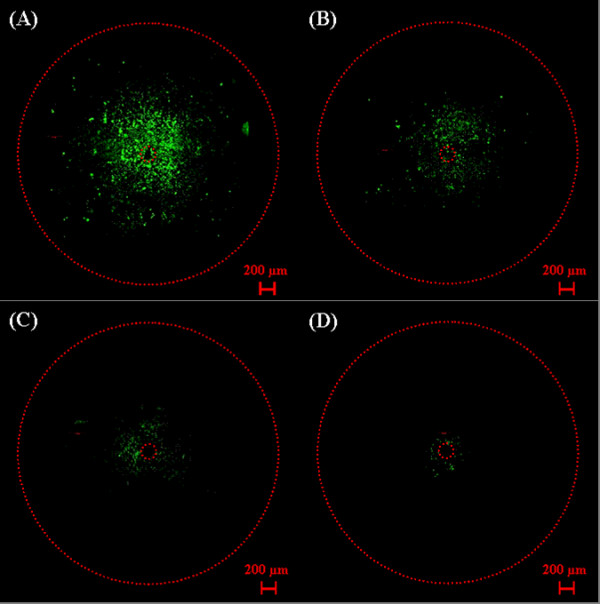
**Fluorescence images of U251 glioblastoma cells [(A) and (C)] and keratoacanthoma cells [(B) and (D)] after nanoelectropulse exposure (1000, 10 kV, 15 ns pulses at 50 Hz) with the flat-cut cable electrode at a distance of < 25 μm [(A) and (B)] and 200 μm [(C) and (D)], respectively**.

Different cell lines may show different sensitivities to the same nanoelectropulse dose with same electrode configuration. To demonstrate this, we applied pulses (1000, 15 ns, 10 kV pulses at 50 Hz) with the flat-cut cable electrode to cell monolayers (U251 glioblastoma and keratoacanthoma) in fresh DMEM containing 1 μM YO-PRO-1. Two different separation distances of 15-20 μm and 200 μm between the monolayer of cells and the electrode tips are applied here. Comparing the two cell lines when the monolayer is 15-20 μm from the tip of the center electrode demonstrates that the U251 glioblastoma cells (Figure 8(A)) are more nanoelectropulse-sensitive than keratoacanthoma cells (Figure 8(C)). When the spacing distance between monolayer cells and electrode increases from 15-20 μm to 200 μm, the influx of YO-PRO-1 into the permeabilized cells decreases for both U251 glioblastoma cells and keratoacanthoma cells shown as Figure 8(B) and Figure 8(D), respectively. The differences in the fluorescence patterns for the U251 glioblastoma and keratoacanthoma cell monolayers indicate that this method can be used to sense the nanoelectropulse-induced permeabilization of various cell types. It also reveals that the electric field distribution changes with different spacing distance. After appropriate calibration, this method may be used for a more detailed exploration of the effects of pulse number, pulse duration, and repetition rate, and of different electrode configurations, on the responses of cell monolayers to nanoelectropulse stimulation.

### 3.4 Thermal effect induced by electric pulses

#### 3.4.1 Experimental measurement

**Figure 9:**
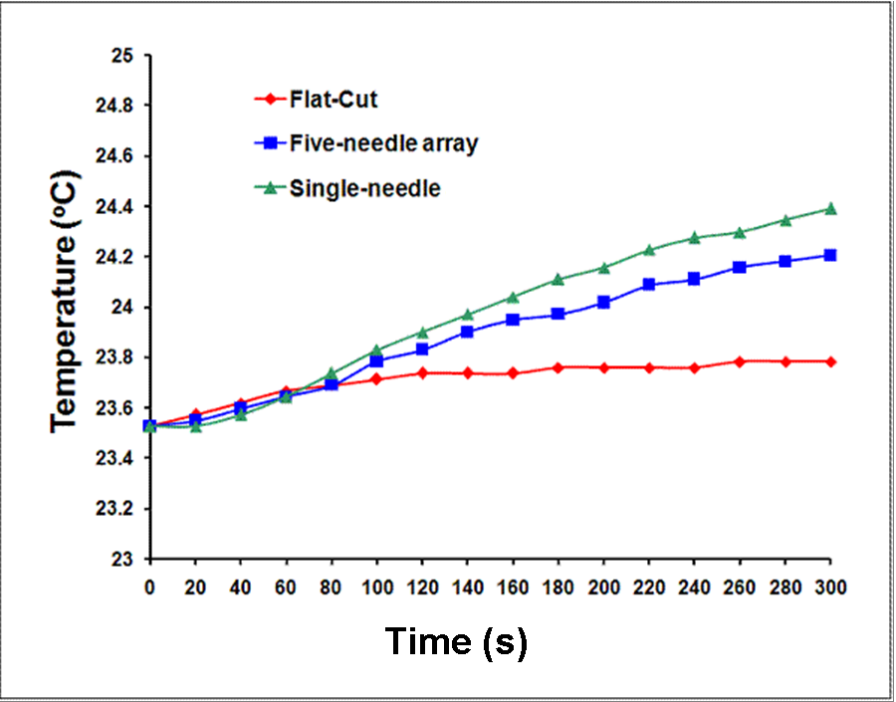
**The temporal development of the temperature at the tips of single-needle, five-needle array, and flat-cut cable electrodes powered with continuous electric pulses (10 kV, 15 ns @ 50 Hz)**.

To exclude possible influence on heat dissipation brought by the RTD as well as the temperature response time (5 seconds), continuous pulses (10 kV, 15 ns @ 50 Hz) were applied for 5 minutes and temperature was recorded every 20 seconds (1000 pulses per data point, 15000 pulses in total). The increases of the localized temperature measured by RTD for three electrode configurations are below 1°C, as shown in Figure 9. The highest temperature increase was found in the single-needle array compared to the five-needle array and the flat-cut electrodes. Table 2 shows measured values of energy per pulse and temperature change for 15000 pulses for each electrode configuration.

**Table 2:** Temperature increase and energy per pulse of three different electrode configurations form measurement.

Electrode Configurations	Energy(mJ)/per pulse	Temperature Change (°C/15 000 pulses)
Single needle electrode	19.1	0.9
Five-needle array electrode	20.5	0.7
Flat-cut cable	1.5	0.3

#### 3.4.2 Numerical calculation

The measurement results suggest that heating induced by nanosecond electric pulses is negligible and is not sufficient to produce permeabilization in cell membranes or to induce hyperthermal effects [18,19]. It can be expected that joule heating produced by each 15 ns pulse is near the surfaces of stainless steel needles and can be completely or partly conducted away with heat diffusion before the 2^nd ^pulse arrives (after 20 ms). For simplicity, we first calculated a model in which a 10 μm gap filled with water between a heat insulator plate at left and a stainless steel plate at right. In addition, considering the thermal diffusivity of stainless steel is 4 × 10^-6 ^m^2^/s, 26 times greater than water, 1.45 × 10^-7 ^m^2^/s, temperature of stainless steel plates can be assumed constant at room temperature, 273 K. For estimation, the 1-dimensional Fourier's equation for heat diffusion was derived by using separation-variable solution. According to 1-dimensional Fourier's equation for heat diffusion

where T(x, t) indicates the temperature distribution, and D is thermal diffusivity of the medium. With separation of variable solution, we obtain

Assuming the initial condition: T (x, 0) = T_0_; 0<x<L, and the boundary condition: T (L, t) = 0; and , for t>0. Equation (4) can be solved to be:

or

where , and *τ*_0_/*τ*_1 _= 9 (*τ*_0_>>*τ*_1_).

A time constant,  = 280 μs, can be assumed for the time required to conduct the electric pulse-induced heat away. This means that the time for temperature to drop to one third of maximum temperature (e^-1 ^= 0.368) is 280 μs. After 20 ms, the increased temperature induced by the first electric pulse will be is negligible. For 2D and 3D calculations, we expect the time constants will be in the same order of magnitude. In addition, there are other forms of heat dissipation including convection and radiation which will help equalize the temperature even faster. Therefore, based on the above experimental measurements and thermal conduction calculation, the thermal effect induced by the nanosecond pulses are negligible.

## 4. Conclusion

Electric field distributions for three different electrode configurations have been evaluated based on nanoelectropulse-induced YO-PRO-1 influx and electrostatic models. The measurement method was also used to gauge the electropermeabilization sensitivity of different cell lines. The visualization of the two-dimensional pattern of permeabilization in living cell monolayer allows us to map the electric field distribution with nanoelectropulses in a biological system for different kinds of electrode configurations. More important, we have proved that a diagnostic tool based on electropermeabilization of cells can be used to test invasive, minimum invasive and noninvasive electrodes for nanoelectropulse therapy. This method can be expected to test the sensitivity of tissues from patients, animals or plants to nanoelectropulses for ex-vivo studies. It also has potential to construct a three-dimensional nanosecond electric field distribution mapping by combining a series of fluorescence images taken with sequential spacing distance between the cell monolayers and electrode.
